# Improved Nocturnal Glycaemia and Reduced Insulin Use Following Clinical Exercise Trial Participation in Individuals With Type 1 Diabetes

**DOI:** 10.3389/fpubh.2020.568832

**Published:** 2021-01-07

**Authors:** Olivia McCarthy, Rachel Deere, Max L. Eckstein, Jason Pitt, Ben Wellman, Stephen C. Bain, Othmar Moser, Richard M. Bracken

**Affiliations:** ^1^Applied Sport, Technology, Exercise and Medicine Research Centre (A-STEM), College of Engineering, Swansea University, Swansea, United Kingdom; ^2^Department for Health, University of Bath, Bath, United Kingdom; ^3^Division of Exercise Physiology and Metabolism, Department of Sport Science, University of Bayreuth, Bayreuth, Germany; ^4^Division of Endocrinology and Diabetology, Department of Internal Medicine, Medical University of Graz, Graz, Austria; ^5^Diabetes Research Group, Medical School, Swansea University, Swansea, United Kingdom

**Keywords:** type 1 diabetes (T1D), exercise, insulin, glycaemia, research participant experience

## Abstract

**Aim:** To explore the influence of clinical exercise trial participation on glycaemia and insulin therapy use in adults with type 1 diabetes (T1D).

**Research Design and Methods:** This study involved a secondary analysis of data collected from 16 individuals with T1D who completed a randomized clinical trial consisting of 23-h in-patient phases with a 45-min evening bout of moderate intensity continuous exercise. Participants were switched from their usual basal-bolus therapy to ultra-long acting insulin degludec and rapid-acting insulin aspart as well as provided with unblinded interstitial flash-glucose monitoring systems. To assess the impact of clinical trial participation, weekly data obtained at the screening visit (pre-study involvement) were compared against those collated on the last experimental visit (post-study involvement). Interstitial glucose [iG] data were split into distinct glycaemic ranges and stratified into day (06:00–23:59) and night (00:00–05:59) time periods. A *p*-value of ≤ 0.05 was accepted for significance.

**Results:** Following study completion, there were significant decreases in both the mean nocturnal iG concentration (Δ-0.9 ± 4.5 mmol.L^−1^, *p* < 0.001) and the time spent in severe hyperglycaemia (Δ-7.2 ± 9.8%, *p* = 0.028) during the night-time period. The total daily (Δ-7.3 ± 8.4 IU, *p* = 0.003) and basal only (Δ-2.3 ± 3.8 IU, *p* = 0.033) insulin dose requirements were reduced over the course of study involvement.

**Conclusions:** Participation in clinical research may foster improved nocturnal glycaemia and reduced insulin therapy use in people with T1D. Recognition of these outcomes may help encourage volunteers to partake in clinical research opportunities for improved diabetes-related health outcomes.

**Clinical Trial Registration:** DRKS.de; DRKS00013509.

## Introduction

Volunteering as a research participant displays altruism and a willingness to help advance medical science. However, research trial participation often requires unaccustomed adjustments to routine care, as well as considerable time commitments for those involved. As such, patient enrollment, and indeed retention, represent long-standing obstacles in the conduction of clinical research (1–3). Nevertheless, research participation can provide patients access opportunities to novel pharmacological therapies and/or technological devices, as well as intense and frequent interactions with clinical research teams who provide educational support. For individuals with type 1 diabetes (T1D), pharmaceutical developments in modern ultra-long acting basal insulins with refined pharmacokinetic and pharmacodynamic profiles have led to improved glycaemic outcomes (4–10). Furthermore, recent advances in interstitial glucose (iG) monitoring systems have challenged the sole dependency on self-monitoring of blood glucose, and proven useful in aiding patient adherence to frequent glycaemic assessment (11–14). In addition, interactive opportunities with health care professionals who offer medical information and support can foster positive psychosocial and glycaemic outcomes (15–17). These pharmaceutical, technological, and physiological aids are complemented by lifestyle factors, including both diet and physical activity. Though exercise is endorsed by several international consensus panels as an integral component of the treatment plan of those with T1D (18–21), participation rates remain low, with fears around loss of glycaemic control and uncertainty in how to appropriately adjust exogenous insulin therapy cited as leading factors dissuading regular engagement (22). The heightened bioenergetic demands of exercising muscle can induce increases in intramuscular glucose uptake by up to 50-fold that of basal rates (23, 24). When combined with an inability to lower exogenous insulin concentrations as well as an often blunted glucoregulatory rescue system (25), the maintenance of normoglycaemia during exercise is challenging for those with T1D. Though acutely apparent, the metabolic challenges evoked by physical exercise may persist for several hours subsequent to its cessation (26–29). This often extends the risk of dysglycaemia leading into and throughout the nocturnal period, at a time when self-monitoring of blood glucose is inherently difficult. As such, glycaemic management strategies that seek to address these concerns are integral in encouraging safe exercise performance whilst minimizing the extent of glycaemic fluctuations. The ideal therapeutic care of those with T1D involves a multimodal approach including access to current pharmacological, technological, and support opportunities that collectively help to cultivate optimal self-management. Thus, research trials that include any of these elements may have clinically relevant outcomes beyond the those solely pertinent to answering the primary outcome.

### Aim

To explore the influence of clinical exercise trial participation on glycaemic and insulin therapy outcomes in adults with type 1 diabetes (T1D).

## Methodology

### Study Design

This study was a secondary analysis of data collected from a single-centered, randomized, open-label, four-period, cross-over clinical trial (DRKS.de; DRKS00013509) consisting of four 23-h in-patient phases with a 45-min evening bout of semi-recumbent cycling at 60 ± 6% VO_2max_. The study was performed in accordance with good clinical practice and the Declaration of Helsinki (https://www.wma.net/policies-post/wma-declaration-of-helsinki-ethical-principles-for-medical-research-involving-human-subjects/). Approval was granted by both the national research ethics committee (16/WA/0394) and the local health authority (EudraCT number: 2017-004774-34; UTN: U1111-1174-6676). The primary outcome was to detail the extent and prevalence of post-exercise and nocturnal hypoglycaemia following peri-exercise bolus insulin dose adjustments in individuals with T1D using multiple daily injections of insulins aspart (IAsp) and degludec (IDeg). As part of a secondary, retrospective analysis, the present study sought to explore the influence of clinical exercise trial participation on glycaemia and insulin therapy use in adults with T1D.

### Screening Visit

Ahead of trial inclusion, participants were screened for anthropometric, cardiovascular, and T1D specific markers prior to the performance of a cardio-pulmonary exercise test on a semi-recumbent cycle ergometer (Corival Recumbent, Lode, NL) (30). Main inclusion criteria were: diagnosis of T1D for ≥12 months; age 18–65 years (both inclusive); body mass index of 18.0–29.4 kg.m^2^; use of multiple daily injections of insulin for ≥12 months; body mass-specific peak oxygen uptake of ≥20 mL.kg^−1^.min^−1^, and a status of being physically active as assessed by the International Physical Activity Questionnaire Short Form. All participants were considered hypoglycaemic aware, having avoided recurrent severe hypoglycaemia (defined as >1 severe hypoglycaemia event during the previous 12 months) and demonstrated a sound understanding of the symptomatic traits of hypoglycaemia to the investigator. After successful completion against the reference inclusion criteria, participants were switched from their usual basal/bolus insulin therapies (*n* = *8*; glargineU100/IAsp, *n* = *1*; glargineU300/IAsp, *n* = *1*; IDeg/IAsp, *n* = *6*; detemir/IAsp) to ultra-long-acting IDeg (Tresiba®, NovoNordisk, Denmark) in 3 mL pre-filled investigational pens (PDS290) and rapid-acting IAsp (NovoRapid® NovoNordisk, Denmark) in 3 mL pre-filled investigational pens (FlexPen®). Therapy with IDeg began on the morning following trial inclusion with a starting dosage of 70–80% of total daily basal insulin dose (TDBD) calculated by means of a titration algorithm. Participants were required to achieve a mean overnight-fasted morning blood glucose (BG) value of 4.4–7.2 mmol.L^−1^ over 3 consecutive days within 4 weeks of the first basal insulin dose. If glycaemic instability persisted for ≥3 days following titration, a dose adjustment alteration was made until criteria was met. A run-in period of >7 days was required to assure optimal adaptation to IDeg prior to the experimental period.

Unblinded flash glucose monitoring readers and sensors (Freestyle® Libre, Abbott, Lake Bluff, Illinois, USA) were provided by the study site for the duration of the trial. The sensor was inserted into the posterior aspect of the upper arm and measured interstitial glucose (iG) in 15-minute intervals. Participants were trained in use of the system and asked to change the sensor at least 48 h before each trial visit to avoid sensor expiration during the research period. With the exception of one individual, all participants were new to use of interstitial glucose monitoring, having previously used a range of point of care self-blood glucose monitoring systems. Though familiar with carbohydrate counting and insulin: carbohydrate dosing ratios, participants were guided through how to accurately record dietary information and shown insulin dose adjustment algorithms by the research team. This included the calculation of their individualized carbohydrate ([CarbF] = 5.7^*^kg/total daily dose of insulin [TDD]) and correction ([CorrF] = 109 mmol/l/TDD) factors as previously described (31). For the remainder of their involvement, participants were monitored by the study personnel to ensure glycaemic stability prior to each experimental visit. Stability was assessed via inspection of their iG patterns with particular scrutiny in the avoidance of hypoglycaemia (≤3.9 mmol.L^−1^) prior to laboratory attendance. As to control for any potential influence of extraneous variables on experimental trial day activities, participants were asked to replicate their habitual diet, physical activity, and insulin dosing strategies in the 24 h prior to each laboratory visit. Participants were contacted frequently by the research team to provide details of any adjustments.

### Experimental Visits

Following preliminary testing (visit 1) and a run-in period for adjustment to IDeg, visits 2, 3, 4, and 5 were experimental visits that involved 23-h of in-patient monitoring with an overnight stay in a clinical research facility. After a standardized day-time period (08:00–15:59), participants undertook a bout of evening (17:00) cycling exercise at 60 ± 6 % $\overset{∙}{V}$O_2max_ One hour prior to, and following exercise, participants administered either a full (100%) or reduced (50%) dose (100%; 5.1 ± 2.4 vs. 50%; 2.6 ± 1.2 IU, *p* < 0.001) of individualized IAsp alongside the consumption identical low-glycaemic index carbohydrate (CHO) rich meals (1.0 g.CHO.kg^−1^). An unaltered and regular dose of IDeg was kept consisted across each experimental visit. Trial day glycaemia was determined via capillary (08:00–15:59), venous (16:00–07:00), and interstitial (08:00–07:00) glucose monitoring over the 23-h in-patient stays.

### Pre vs. Post Study Data Analysis Methodology and Statistical Analysis

**Figure 1:**
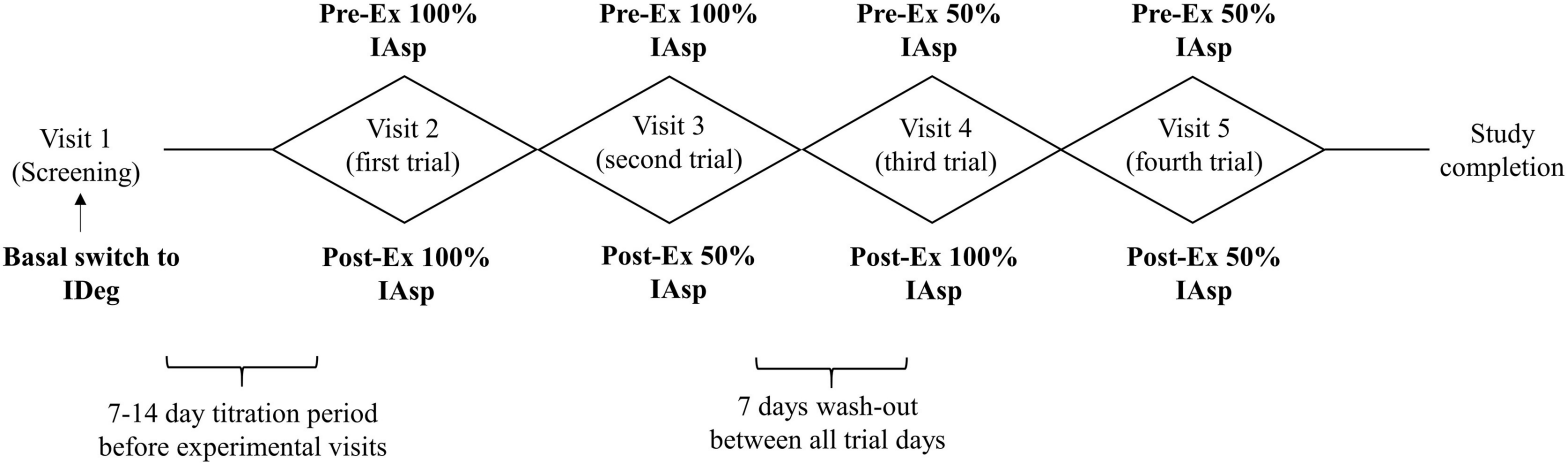
Schematic overview of the trial design with reference to the four interventional cross over arms involving peri-exercise adjustments to bolus insulin aspart on a stable background of insulin degludec. IAsp, insulin aspart; IDeg, insulin degludec.

Over the course of the study it became apparent that many individuals were perceiving their participation experience as beneficial to aspects of their diabetes related care outside of the experimental periods. Thus, as part of a retrospective, observational, secondary analysis we investigated weekly data taken from a “pre-study” period and compared them against data taken in the final week of their enrolment i.e., “post-study.” The pre-study period was classified as the 6-days after the screening visit but before any experimental trial visits (between visits 1 and 2), whilst the post-study period was classified as the 6-days prior to the final experimental trial visit immediately ahead of study completion (between visits 4 and 5). A 6-, rather than 7-day average was taken to avoid any potential interference of trial-related activities on habitual behaviors. Figure 1 provides a schematic overview of the study design with reference to the primary interventional manipulations and experimental visit schedule.

On trial days, participants intake weighed whilst fasted and asked to report their previous 6-day average basal and bolus insulin doses, CHO intake and physical activity patterns (International Physical Activity Questionnaire). iG data were stratified into time spent within glycaemic thresholds i.e., time in range (TIR), time below range (TBR) and time above range (TAR). The targets are further bracketed into levels 1 and 2 to detail the severity of both hypo-and hyper-glycaemia; TBR 2 (<3.0 mmol.L^−1^), TBR 1 (≥3.0– <3.9 mmol.L^−1^), TIR (≥3.9– ≤ 10.0 mmol.L^−1^), TAR 1 (≥10.1– ≤ 13.9 mmol.L^−1^), TAR 2 (>13.9 mmol.L^−1^) (32). iG data were also split into day (06:00–23:59) and night (00:00–05:59) time periods. Due to a significant loss of data points, four participants were excluded from iG analysis. The significance of change in measurements from pre- to post-study was assessed via paired student's *t*-test or non-parametric equivalents used when necessary. SPSS (version 26.0) was used for all data analyses and reporting. *P* values of a *p* ≤ 0.05 (two-sided) accepted as statistically significant.

## Results

### Participant Characteristics

Baseline characteristics of study participants are included in Table 1. The average length of time for trial participation was 55 ± 29 days.

**Table 1:** Baseline characteristics of study participants.

**Baseline characteristics of study participants**	
**Characteristic**	***n=*** **16**
Gender M vs. F (*n*)	13 vs. 3
Age (years)	34.5 ± 13.9
BMI (kg/m^2^)	26.0 ± 3.4
Lean mass (%)	23.4 ± 3.3
HbA_1c_ (%)	7.2 ± 1.3
HbA_1c_ (mmol/mol)	56 ± 15
Diabetes duration (years)	14.4 ± 11.1
Pre study TDD (IU)	51.7 ± 26.6
Pre study TDBD (IU)	31.3 ± 21.3
*V°*O_2max_ (ml.kg^−1^.min.^−1^)	40.3 ± 10.3

*Data are presented as mean ± SD. n, number of participants*.

*M, Male; F, Female; BMI, body mass index; Kg, kilograms; M, meters; TDD, Total daily insulin dose (inclusive of basal and bolus amounts); TDBD, total daily basal insulin dose; Bm, body mass; ml, millimeters; Min, minutes*.

### Interstitial Glucose Outcomes

Device coverage was ≥89% over the 6-day data capture in both the pre- and post-study phases (pre; 91 ± 19 vs. post; 89 ± 14%, *p* = 0.716). Overall and stratified iG are presented in Table 2. Analysis revealed significant decreases in both the mean nocturnal iG concentration (Δ-0.9 ± 4.5 mmol.L^−1^, *p* < 0.001) and the TAR2 (Δ-7.2 ± 9.8%, *p* = 0.028) during the night-time hours.

**Table 2:** Time spent in each glycaemic range as part of a 6-day analysis from the first to last experimental trial visits.

**Parameter**	**Pre-study**	**Post-study**	***P*-value**
Overall glucose (mmol.L^−1^)	9.23 ± 4.38	9.07 ± 4.30	0.355
Standard deviation (mmol.L^−1^)	3.83 ± 0.91	3.84 ± 1.02	0.965
CoV (%)	40.99 ± 4.42	42.21 ± 7.37	0.598
**Stratified (24 h)**			
Overall TBR2 (%)	2.83 ± 3.91	3.84 ± 2.29	0.246
Overall TBR1 (%)	3.97 ± 1.90	4.00 ± 3.08	0.971
Overall TIR (%)	55.28 ± 18.80	55.26 ± 13.99	0.995
Overall TAR1 (%)	22.15 ± 8.33	23.87 ± 9.82	0.571
Overall TAR2 (%)	15.77 ± 14.74	13.02 ± 10.55	0.391
**Stratified Day; 06:00–23:59 vs. Night; 00:00–05:59**			
Day glucose (mmol.L^−1^)	9.03 ± 4.38	9.15 ± 4.18	0.270
Day TBR2 (%)	2.55 ± 4.35	3.25 ± 3.08	0.495
Day TBR1 (%)	4.58 ± 2.02	4.20 ± 3.64	0.701
Day TIR (%)	57.06 ± 19.59	56.79 ± 14.62	0.954
Day TAR1 (%)	21.60 ± 8.16	23.19 ± 9.31	0.589
Day TAR2 (%)	14.22 ± 14.18	12.57 ± 10.26	0.662
Night glucose (mmol.L^−1^)	9.84 ± 4.52	8.98 ± 4.48	<0.001^*^
Night TBR2 (%)	3.23 ± 5.64	5.13 ± 5.31	0.138
Night TBR1 (%)	2.00 ± 3.10	3.43 ± 3.62	0.367
Night TIR (%)	50.06 ± 21.18	52.06 ± 16.56	0.673
Night TAR1 (%)	24.22 ± 14.30	26.02 ± 11.57	0.748
Night TAR2 (%)	20.54 ± 18.65	13.35 ± 16.63	0.028^*^

*CoV, coefficient of variation; TBR 2, time below range level 2 (<3.0 mmol.L^−1^); TBR 1, time below range level 1 (≥3.0– <3.9 mmol.L^−1^); TIR, time in range (≥3.9– ≤ 10.0 mmol.L^−1^); TAR 1, time above range level 1 (≥10.1– ≤ 13.9 mmol.L^−1^); TAR 2, time above range level 2 (>13.9 mmol.L^−1^)*.

* *p ≤ 0.05 between first pre- and post-study values. Data are reported as mean ± SD. n =12*.

### Anthropometry and Insulin Therapy Outcomes

There were significant reductions in both the total daily (Δ-7.3 ± 8.4 IU, *p* = 0.003) and basal only (Δ-2.3 ± 3.8 IU, *p* = 0.033) insulin dose requirements from pre-to-post-study involvement (Table 3). There were no changes in any of the anthropometric, dietary CHO or physical activity metrics.

**Table 3:** Participant insulin regime, anthropometric data, and physical activity patterns on the first vs. last trial visits.

**Weekly data from pre-to post-study involvement**			
**Parameter**	**Pre-study**	**Post-study**	***P-*****value**
TDD (IU)	51.7 ± 26.6	44.4 ± 20.7	0.003^*^
TDBD (IU)	31.3 ± 21.3	29.0 ± 18.4	0.033^*^
Body mass (kg)	80.0 ± 9.9	80.1 ± 9.6	0.785
CHO intake (g)	194.2 ± 58.0	190.8 ± 64.5	0.666
Physical activity (METs)	3600.3 ± 2943.7	3359.7 ± 2491.9	0.678

*TDD, Total daily insulin dose (inclusive of basal and bolus); TDBD, Total daily basal insulin dose; IU, International units; CHO, carbohydrates; METs, metabolic equivalents*.

* *p ≤ 0.05 between pre- and post-study values. Data are presented as mean ± SD*.

## Discussion

This exploratory study investigated the wider glycaemic impact of participation in a clinical exercise trial involving a therapeutic switch to ultra-long acting insulin degludec as well as the introduction to, and *ad-hoc* education support with, an interstitial glucose monitoring system in individuals with T1D.

Comparative analysis of interstitial glucose (iG) data obtained over a 6-day period taken before vs. after clinical trial participation revealed significant reductions in both the mean nocturnal glucose concentration and the amount of time spent in severe hyperglycaemia during the night-time hours. Additionally, upon trial completion, substantial reductions in both total daily, and basal only, insulin dose requirements were noted in the absence of changes in any anthropometric, dietary, and physical activity factors.

The reduction in insulin dosing requirements in the present study aligns with previous investigations that have demonstrated the efficacy of IDeg therapy in maintaining glycaemic outcomes at significantly lower dosing amounts (33, 34). The end of study IDeg dosing quantities used in this study are similar to those reported in recent work by Heise et al. (9) (Heise; 0.38 ± 0.23 IU.kg^−1^ vs. Our data; 0.34 ± 0.20 IU.kg^−1^), which reaffirms the safe integration of IDeg as a stable basal therapy at clinically relevant dosing levels. These dose reductions occurred in the absence of any changes in body mass, carbohydrate intake and physical activity patterns. Though we cannot out rule the possibility that great diligence to dietary tracking may have triggered individuals to select healthier food options, including lower glycaemic index carbohydrates, which may have contributed to drop in insulin dose, in light of the potential obesogenic implications associated with an over reliance on exogenous insulin administration (35), the dose reductions observed in this study carry important clinical undertones that stretch beyond those relating to dysglycaemia. Furthermore, 14/15 (93%) participants opted for continued use of IDeg as their basal analog and applied locally for continued Freestyle Libre provision upon study completion, perhaps emphasizing the value of these therapeutics in terms of patient satisfaction.

The significant decreases in both mean iG concentrations and the amount of TAR2 during nocturnal hours are meaningful from both a practitioner and patient point of view. Given the lack of endogenous autoregulation in the synthesis and secretion of insulin, the prevalence of the dawn phenomenon is a common feature of T1D which continues to represent a serious clinical concern (36). Combined with the inherent difficulties of performing regular self-monitoring of blood glucose during sleep, dysglycaemia during the night-time constitutes a major worry not only for those with T1D, but also for those who take an active role in their care (37–39). These fears are perhaps magnified following evening exercise, which, due to its long-lasting insulin-sensitizing effects, can disrupt glycaemia for the many hours subsequent to its performance (29, 40–42). Several international panels of diabetes specialists have convened to outline the merits of utilizing iG metrics to support decision making in clinical care (32, 43). The improvements in nocturnal glycaemia observed in this study may be the result of the introductory provision of an iG monitoring device, which allowed for the assessment of glycaemic patterns throughout the night-time period and hence, the ability to act accordingly to prevent glycaemic excursions. These results offer encouragement for the integration of modern ultra-long acting insulin analogs alongside iG monitoring systems in aiding glucose management around physical exercise in those with T1D. An important caveat is that our study cohort had a relatively long diabetes duration (~14 years), thus may have had greater experience in glycaemic management around physical exercise than those with a newer diagnosis.

For those with T1D, the fear of hypoglycaemia around exercise prevails as the main barrier to regular engagement, whilst a greater knowledge of insulin pharmacokinetics and/or using appropriate approaches to minimize exercise-related hypoglycaemia are associated with fewer perceived hurdles (22). The volatility in blood glucose levels around exercise may be one of the reasons that >60% of individuals with T1D currently fail to meet physical activity guidelines (44). To that end it is encouraging to learn of the potential value interactions with health care professionals and exercise physiologists throughout trial participation may have on the individuals involved, who's willingness to participate in clinical trials help advance our research efforts.

Due to the provision of two therapeutic aids alongside access to clinical diabetes care and support, it is difficult to discern the exact source of the observed improvements. Rather, we put forth these findings as part of a multi-faceted analysis, that, irrespective of being able to irrefutably demonstrate a clear cause and effect relationship, highlights the beneficial effects of research participation in a mutually reciprocal manner. Recognition of these outcomes may help incite volunteers to partake in clinical trials as well as encourage scientists to explore hypotheses outside of the primary objective.

## Conclusion

Participation in clinical research may foster improved nocturnal glycaemia and reduced insulin therapy use in people with T1D. Beyond pursuing the primary outcomes of a research hypothesis, these data provide a basis for exploring the wider, clinically relevant health outcomes that may be associated with research trial participation.

## Data Availability Statement

The raw data supporting the conclusions of this article will be made available by the authors, without undue reservation.

## Ethics Statement

The studies involving human participants were reviewed and approved by the national research ethics committee (16/WA/0394) and the local health authority (EudraCT number: 2017-004774-34, UTN: U1111-1174-6676). The patients/participants provided their written informed consent to participate in this study.

## Author Contributions

OMc, OMo, RD, ME, JP, and RB were responsible for data collection. OMc, JP, BW, and RB were responsible for data interpretation and statistical analyses. OMc, JP, and RB wrote the manuscript. SB and RB were the chief and principle investigators of the study. SB provided medical oversight for the study. RB wrote and secured funding for the study. All authors contributed to feedback and revisions for the final manuscript.

## Conflict of Interest

The authors declare that this study was funded by Novo Nordisk A/S as part of an investigator sponsored study. The funders had no role in study design, data collection and analysis, decision to publish, or preparation of the manuscript. OMc* has received a Zienkiewcz scholarship and travel grants from Novo Nordisk. OMo has received lecture fees from Medtronic, travel grants from Novo Nordisk A/S, Novo Nordisk AT, Novo Nordisk UK and Medtronic AT, research grants from Sêr Cymru II COFUND fellowship/European Union, Sanofi-Aventis, Novo Nordisk A/S, Novo Nordisk AT, Dexcom Inc., as well as material funding from Abbott Diabetes Care. ME has received a KESS2/European Social Fund scholarship and travel grants from Novo Nordisk A/S and Sanofi-Aventis. SB has received research grants (includes principal investigator, collaborator or consultant and pending grants as well as grants already received) from Health care and Research Wales (Welsh Government) and Novo Nordisk. He has received other research support from Healthcare and Research Wales (Welsh Government), honoraria from Novo Nordisk, Sanofi, Lilly, Boehringer Ingelheim and Merck, and has an ownership interest in Glycosmedia (diabetes on-line news service). RB reports having received honoraria, travel, and educational grant support from Boehringer-Ingelheim, Eli Lilly and Company, Novo Nordisk, and Sanofi-Aventis. The remaining authors declare that the research was conducted in the absence of any commercial or financial relationships that could be construed as a potential conflict of interest.
